# Spin–Orbit Torque in Single-Molecule Junctions from *ab Initio*

**DOI:** 10.1021/acs.jpclett.4c00502

**Published:** 2024-05-22

**Authors:** María Camarasa-Gómez, Daniel Hernangómez-Pérez, Ferdinand Evers

**Affiliations:** † Institute of Theoretical Physics, University of Regensburg, 93040 Regensburg, Germany; ‡ Department of Molecular Chemistry and Materials Science, Weizmann Institute of Science, Rehovot 7610001, Israel; § CIC nanoGUNE BRTA, Tolosa Hiribidea 76, 20018 San Sebastián, Spain

## Abstract

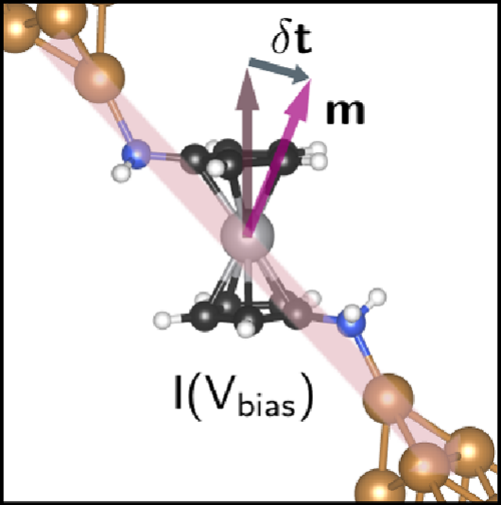

The use of electric fields applied across magnetic heterojunctions that lack spatial inversion symmetry has been previously proposed as a nonmagnetic means of controlling localized magnetic moments through spin–orbit torques (SOT). The implementation of this concept at the single-molecule level has remained a challenge, however. Here, we present first-principles calculations of SOT in a single-molecule junction under bias and beyond linear response. Employing a self-consistency scheme invoking density functional theory and nonequilibrium Green’s function theory including spin–orbit interaction, we compute the change of the magnetization with the bias voltage and the associated current-induced SOT. Within the linear regime our quantitative estimates for the SOT in single-molecule junctions yield values similar to those known for magnetic interfaces. Our findings contribute to an improved microscopic understanding of SOT in single molecules.

The controlled manipulation of magnetic moments in solid state heterojunctions by driving a spin-polarized electrical current is an established technique in spintronics^1^ since its theoretical prediction almost three decades ago^2,3^ and its subsequent experimental observation.^4,5^ This type of spin-transfer torque (STT)^6,7^ can be used to produce current-induced magnetization reversal even at room temperature^7−12^ by transfer of spin angular momentum between the charge carriers and the localized magnetic moments; applications include STT random-access memories.^13^ A potential drawback of STT-based technology is the involvement of high density spin-polarized currents.^14^ Such currents require balancing of magnetoresistance, cross section, and bias voltage, which is believed to be challenging for downsizing devices.^15,16^ Moreover, spin-polarized contacts can also affect localized magnetic moments, as it is well-known in scanning-tunneling microscope setups.^17^

A promising effect that can produce current-induced magnetization switches all electrically, i.e., without invoking spin-polarized driving currents,^13,16,18−24^ is the spin–orbit torque^15,16,25−27^ (SOT); here, the exchange of angular momentum between a local magnetization and the driving current is mediated by spin–orbit (SO) interaction. SOT on localized moments can be produced in systems lacking structure inversion symmetry.^28,29^ Furthermore, due to the potentially small current densities required, it opens up the opportunity to scale down devices to the molecular or even atomic level.

In this work, we devise a self-consistent theoretical scheme which incorporates SO interaction in order to study the SOT in a molecular junction. The simulation method we propose extends previous studies that have confined themselves to the linear regime in the applied electric field through a Kubo-formula based calculation of the torkance tensor,^30−33^ or in which the density matrix is not calculated in a fully self-consistent manner^34−39^ and is therefore limited to the linear response regime in the applied voltage. Similar to these earlier studies, we also adopt the adiabatic approximation, in which the Hamiltonian is assumed to be time-independent.^40^ Our nonequilibrium Green’s function formalism (NEGF) approach is based on density functional theory (DFT); it therefore incorporates details of the electronic structure and geometry, thus extending as well previous model-based investigations.^28,41^

As a case study, we apply our simulation method to a vanadocene–copper molecular junction, as shown in Figure 1. Vanadocene is an open-shell metallocene,^42^ formed by a vanadium atom “sandwiched” between two carbon-based cyclopentadienyl (Cp) rings in a double decker structure. Some of the compounds of this family have recently sparked a renewed interest in view of their intrinsic quantum interference^43^ and light-induced properties.^44^ We find that all electrical control of the localized magnetic moment can be achieved. Within the linear regime, we extract from our results values for the torque that are quantitatively similar to the previous estimates based on the Kubo-formula.

**Figure 1:**
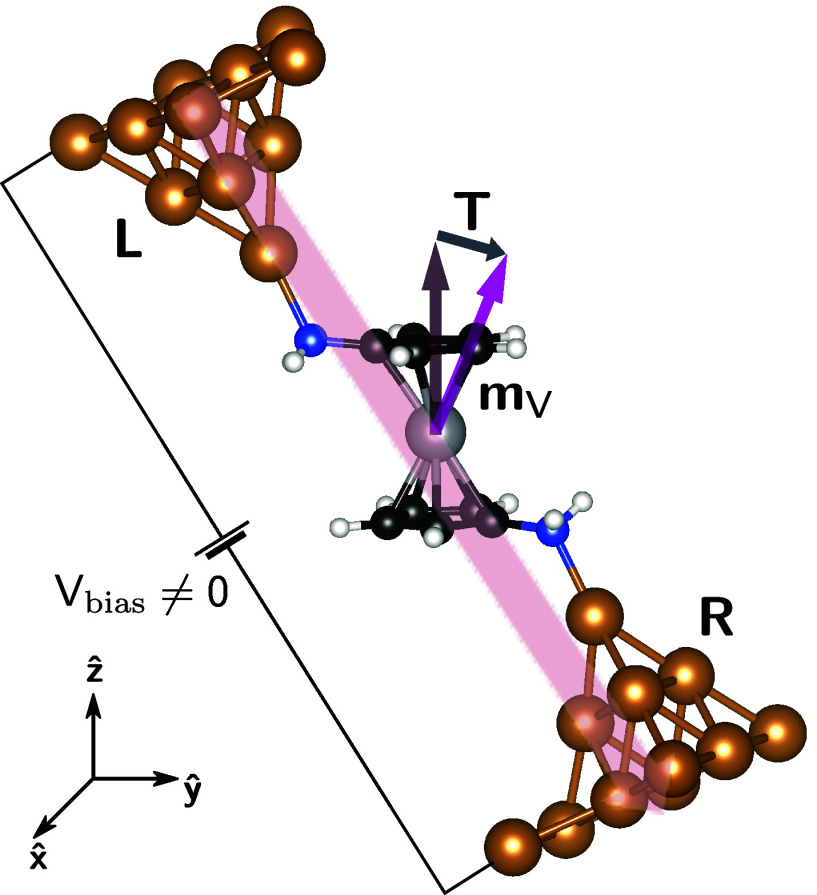
Schematic structure of the molecular junction. As a response to a voltage bias, *V*_bias_, the current, represented by a semitransparent red arrow, flows from the left (L) to the right (R) contact. The magnetic moment, located mostly at the vanadium atom and labeled by **m**_*V*_, reacts and tilts by means of a SOT, **T**, associated with the current flow.

*Model.* Our simulations operate within the framework of noncollinear DFT with a corresponding Kohn–Sham Hamiltonian^34,45−48^
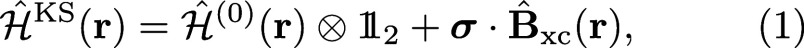
1with **σ** the vector of Pauli matrices, **σ** = (σ_*x*_, σ_*y*_, σ_*z*_); 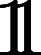
_2_ the 2 × 2 identity matrix,  the spin-independent part of the Kohn–Sham Hamiltonian, and  the exchange-correlation (XC) magnetic field embodying the effect of SO coupling. The XC magnetic field is formally given by the functional derivative of the XC energy with respect to the magnetization, .

*Nonequilibrium Formalism.* Using the Kohn–Sham states, |Ψ_*l*_⟩, and energies, ϵ_*l*_, of eq 1 we build the nonequilibrium density matrix,  following the general construction rules as formulated in refs (49−51). The finite-bias expectation values of observables, , are calculated as usual, i.e., by evaluating the trace . This way we have for the total particle number, , the (spin) magnetization,  [with Bohr’s magneton μ_*B*_ = *eℏ*/(2*m*)] and for the SOT
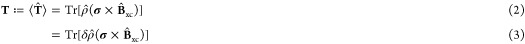
where . The second line reflects the fact that in equilibrium by definition all physical observables are stationary and therefore the SOT has to vanish,  (“zero-torque theorem”^45,52^).^53^ We further mention that the form of the operator  reflects the rate of change of the spin density due to the local exchange-correlation field created by the SO interaction,  where .^34^ Indeed, using Heisenberg’s equation of motion, one has
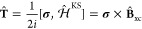
4

*Self-Consistent Simulation Procedure.* We display in Figure 2 the nonequilibrium self-consistency cycle; the technical details have been relegated to the Supporting Information (SI). In short, starting from an optimized geometry of the molecular junction obtained with the FHI-aims package,^54^ we perform a self-consistent DFT^55,56^-NEGF^57,58^ cycle that accounts for the charge redistribution in the junction due to the macroscopic nature of the contacts.^49,59−62^ We ensure the charge neutrality and screening the excess charge accumulated at the boundaries of the finite cluster by a self-energy model implemented in the module AITRANSS.^49,50,59^ This module was recently extended to include SO coupling.^60^ Our self-consistent scheme thus expands available DFT-NEGF self-consistent cycles.^37,38,59,63−65^

**Figure 2:**
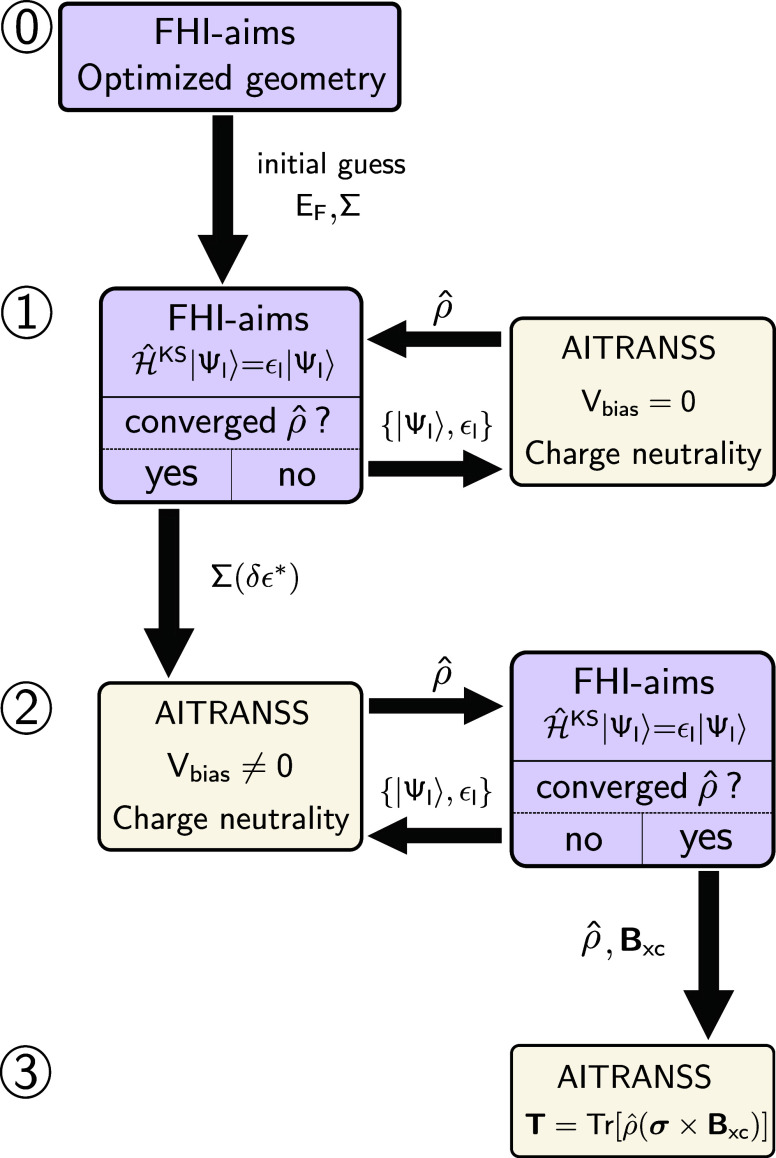
Schematic of the self-consistent cycle considered in this work. The calculation proceeds in four sequential steps (labeled from ⓪−③). After an initial geometry optimization in step ⓪, the self-energy, Σ, and the Fermi energy, *E*_F_, are parametrized in step ①. The optimal self-energy for a given Fermi energy and real self-energy shifts, Σ(δϵ*), is used in the second self-consistent loop at finite bias voltage, see step ②. A further postprocessing step allows physical observables to be computed with the optimized nonequilibrium density matrix, see step ③. Additional details of the cycle are described in the SI.

Typically, the spin carried by the molecular junction implies only a weak magnetic anisotropy energy (MAE) and, as a consequence, the self-consistent field (SCF) iterations are difficult to converge. As is often done in situations with a weakly broken continuous symmetry, we stabilize the convergence of the scf-cycle by operating with a manually enhanced MAE quantified by a small parameter Δ (see SI for further details); at the end of the calculations, we extrapolate into the limit Δ → 0. Since Δ is small, the (weakly) enhanced MAE does not alter the orbital ordering; the corresponding shift of the Kohn–Sham energies is less than the average level spacing close to the frontier orbitals and therefore negligible.

As a demonstration of our implementation of the SCF cycle for the SOT, we consider the junction Figure 1, i.e., a single vanadocene molecule^66^ between two copper contacts. Compared to the traditional gold contacts used in molecular electronics, copper electrodes recommend themselves due to their weak SO interaction. This choice largely guarantees that SOT occurs predominantly at the molecular level.

*Reference Calculation—Isolated Molecule.* Vanadocene possesses a relatively small SO interaction with uniaxial anisotropy and an easy magnetization axis perpendicular to the plane containing each of the Cp rings. Most of the magnetic moment is located on the vanadium atom and is associated with three unpaired electrons in a *e*_2*g*_ + *a*_1*g*_ configuration.^67^ Consequently, DFT (with the PBE functional^68^) yields 3.0 μ_B_ for the spin magnetic moment in the isolated molecule, with the spin of the molecule oriented in the  direction (see reference frame for the molecule in the junction in Figure 1). Further, the DFT-calculation confirms a weak MAE, smaller than 1 meV, explaining the need to use the MAE enhanced by Δ for stabilization of the SCF cycle when bringing the molecule in contact with the electrodes.

*Transport.* In Figure 3, we show the current–voltage characteristics (*I*–*V*_bias_) of the vanadocene–copper junction obtained after employing the self-consistent cycle in Figure 2. We observe that the *I*–*V*_bias_ is linear up to *V*_bias_ ∼ 40 meV, above which deviations due to higher-order terms in the applied bias are found; we associate them to polarization effects that have been discussed in ref (51). These effects are largely independent of the MAE in the junction. We also display in the inset the zero-bias conductance, , which can be extracted from the d*I*/d*V*_bias_ in the limit *V*_bias_ → 0. Taking the limit  we find  (in units of the conductance quantum, ). This result is consistent with transmission calculations performed for ferrocene (amine-linked) gold molecular junctions.^43^

**Figure 3:**
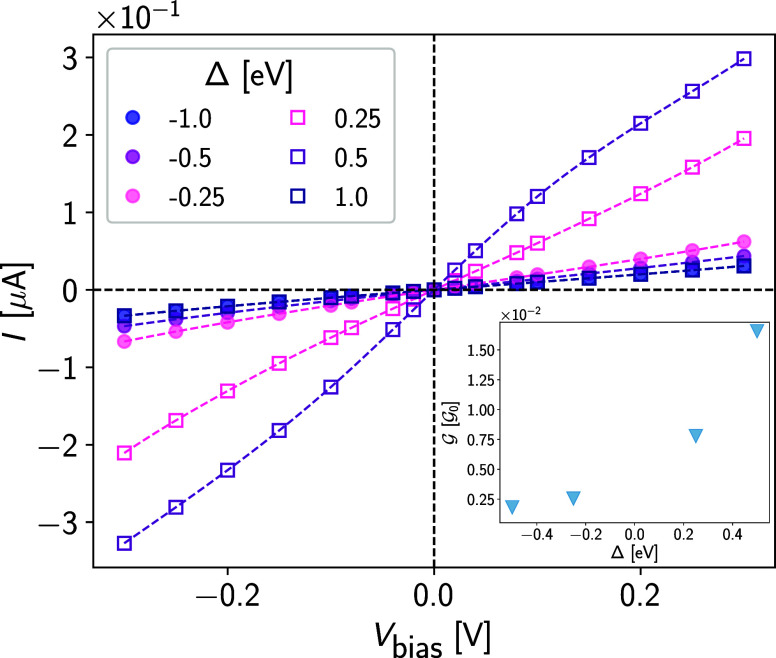
Current–voltage (*I*–*V*_bias_) characteristics of the vanadocene junction for several values of the magnetic anisotropy parameter, Δ. Dashed lines are cubic-spline interpolations. Inset: (Zero-bias) conductance  (in units of the conductance quantum) extracted from the current–voltage characteristics as a function of Δ (dashed lines are guide to the eye) from which the limit  can be inferred.

*Magnetism.* In Figure 4, we display the local magnetic moment, **m**, at the position of the vanadium atom as a function of *V*_bias_. We show the magnetic moment in (a) the  and (b) the  direction (see SI for the  direction) considering as well several values for the magnetic stabilization term, Δ. As a reference, we also display as a dashed horizontal line the value obtained for the local magnetization in the isolated vanadocene molecule, which is directed toward the easy axis ( direction, see Figure 1). We observe first that also after the electrodes have been attached, the magnetic moment is mainly carried by the vanadocene atom, with values close to , and points in the  direction. Also, in both  and  directions, the zero-bias value is largely insensitive to the value of Δ (see inset). We note that *m*_*x*_ exhibits large fluctuations (“noise”) with the applied voltage. Given the small absolute values of the order of 10^–4^ μ_B_, we attribute the noise to limits of the SCF convergence.

**Figure 4:**
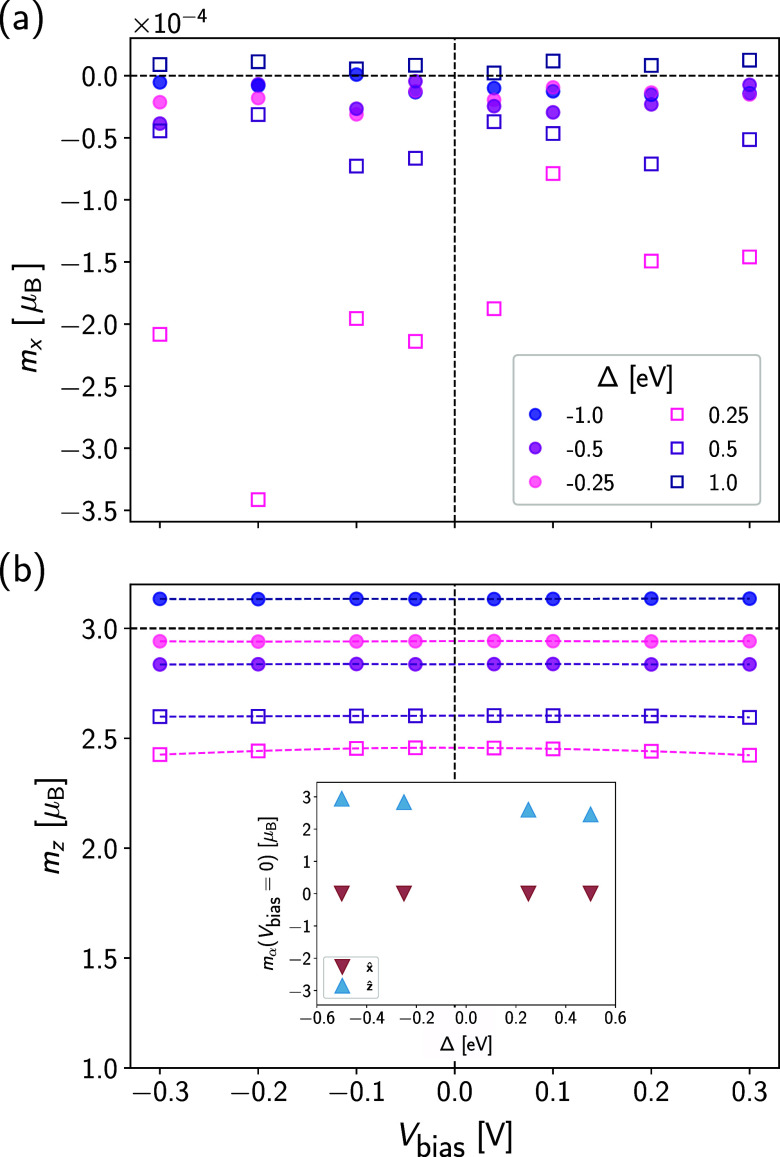
Magnetization, *m*_α_, at the vanadium atom as a function of the voltage bias, *V*_bias_, applied across the molecular junction. We display the traces obtained for several representative values of the magnetic anisotropy term in the two relevant spatial directions (a)  and (b) . The dashed horizontal line represents the value expected for the local magnetization in the isolated vanadocene molecule, oriented in the  direction. The dashed colored lines correspond to cubic-spline interpolations. Inset: Zero-bias magnetization as a function of Δ.

Furthermore, we find that *m*_*z*_ presents a small curvature corresponding to smooth variations of the order of % when the current flows across the molecular junction. In this sense, within the accuracy of the calculation, *m*_*z*_ is not strongly dependent on the current flow because the spin is strongly locked into the easy axis and the SO interaction, which drives the SO torque, is small. We interpret the curvature of *m*_*z*_ with the applied *V*_bias_ as the result of transfer of angular momentum of the itinerant electrons associated with the stationary current flow to the localized spins due to the SO torque.

*Torque.* Similar to the magnetization shown in Figure 4, we display in Figure 5 the local SOT in (a) the  and (b) the  direction (see SI for the  direction) for different values of Δ. We first note that SOT is odd under time reversal and therefore changes sign upon reversal of the bias voltage. This behavior is reflected in simple models employed for heterostructures utilizing the Rashba Hamiltonian in the presence of magnetization;^15^ in these instances it can be demonstrated explicitly that the SOT exhibits a direct proportionality to the current density. Also, the (linear) SOT is seen to be largely insensitive to the magnetic anisotropy parametrized by Δ, so the limit of interest, Δ → 0, is trivial to take. This extrapolation yields very small values for the ratio *δt*_α_/*V*_bias_ in the limit *V*_bias_ → 0. Further, owing to the definition 4, , the magnitudes of torque and magnetization are proportional to each other. Correspondingly, the torque in the directions perpendicular to the magnetic moment exceeds the parallel torque (roughly in -direction, Figure 5) by 2 orders of magnitude.

**Figure 5:**
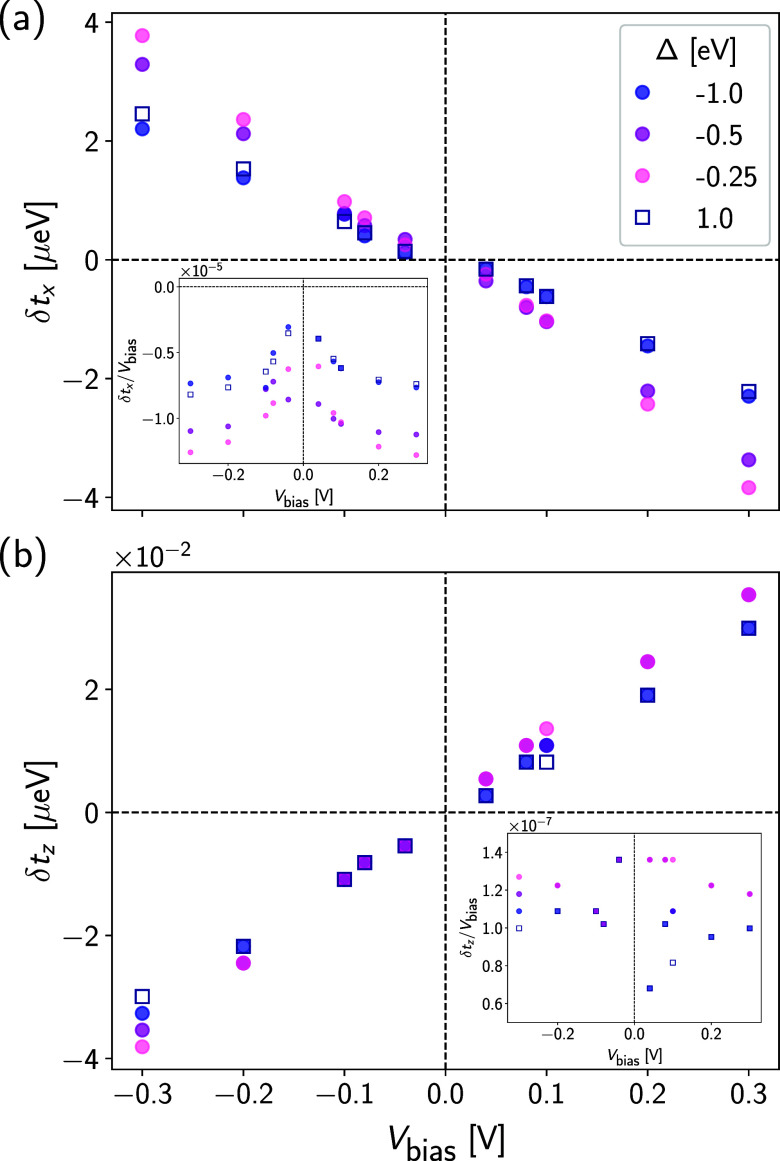
Local SO torque response, *δt*_α_, exerted at the spin located in the vanadium atom as a function of the voltage bias, *V*_bias_, applied across the molecular junction. We display the traces obtained for several representative values of the magnetic anisotropy term in two relevant spatial directions (a)  and (b) . Insets: ratio of the local SO torque response to the bias voltage in the (a)  and (b)  directions.

*Literature Comparison.* In linear response, we have the relation  which introduces the torkance tensor  and the probing electric field **E**; the tensor is well accessible within the conventional linear-response (Kubo-) formalism and has been explored, e.g., in the context of inversion asymmetric magnetic heterostructures.^30,31,69^ For a (semi)quantitative comparison of our results obtained for the vanadium complex with such literature values, we offer a rough estimate:

We first simplify the torkance tensor taking it to be diagonal with two nonvanishing eigenvalues, *t* ≔ *t*_*x*_ = *t*_*y*_ corresponding to the two orthogonal directions  and . We further take the local electric field as orthogonal to the direction  with magnitude *E* ≈ *V*_bias_/*L*, where *L* ∼ 13*a*_0_ is the length of the molecular layer measured as the linear distance between the two anchor groups in units of Bohr’s radius *a*_0_. Figure 3 suggests that the linear response regime extends at least up to bias voltages of *V*_bias_ ∼ 40 meV, corresponding to an electric field *E* ∼ 3 mV/*a*_0_. Considering the ratio *t* = |**T**|/|**E**|, based on Figure 5 we estimate *t* ∼ 3·10^–4^*ea*_0_. For a meaningful comparison with literature values obtained in cobalt (Co) heterostructures,^30^ we rescale the typical torkance values with the typical SO interaction energy, assuming a linear relation between the SOT and the SO interaction strength at lowest order. To estimate the SO interaction strength in the case of the molecular junction, we adopt the typical energy splitting of vanadocene molecular levels due to SO interaction, Δ*E* ∼ 1 meV, as obtained from the Kohn–Sham energies of our DFT calculations (see Table 1 in SI); this results in ξ_*V*_ = *t*/Δ*E* ∼ 0.3*a*_0_/V, for the vanadium-based molecule. For Co-based structures on the other hand, we obtain the rough estimate ξ_Co_ = *t*_Co_/ζ_Co_ ∼ 1.25*a*_0_/V, where we combined a typical value for the torkance in the heterostructure active layer, *t*_Co_ ∼ 0.1*ea*_0_, from ref (30) and a typical energy splitting associated with the SO coupling in a hydrogen-like atom, ζ_Co_ ∼ 0.123 eV from ref (70). As one would expect, the SO-normalized results turned out to be of the same order of magnitude.

*Parasitic Magnetic Fields.* The SOT can be (re)interpreted as an effective (Oersted or coercive) magnetic field, , where  denotes the initial direction of the magnetization before the SOT is applied and μ is the total magnetic moment of the interface molecule.^26,30,71^**B**_coerc_ represents the magnetic field strength that is needed to induce an equivalent effect on the magnetization as the current-induced SOT. Formally, the coercive magnetic field is a function of the applied bias, **B**_coerc_(*V*_bias_), because the SOT depends on the current density. Since in the vanadocene junction , the coercive field is oriented perpendicular to the easy magnetization axis, . For the *x*–*y* plane, the typical value of SOT in the linear response regime is ∼ μeV, which translates into |**B**_coerc_|∼ 0.01 G. With an eye on the production of nanostructured magnetic fields in mesoscopic samples, we mention that this value is 2 orders of magnitude smaller compared to typical current-induced magnetic fields in eddies in graphene sheets.^72^ Therefore, the SOT is likely to compete with unintended (“parasitic”) current-induced magnetic fields that may impair a controlled SOT-mediated switching process, unless a system with large-enough SO coupling has been chosen.

We have presented a self-consistent calculation of SOT in a vanadocene–copper molecular junction at finite bias and in the steady-state regime. Our findings are as follows: (a) The window of linear responses of current and torque is wide and extends beyond 300 meV. (b) Within this voltage window, the local magnetization being located near the vanadium atom is largely insensitive to the current flow. (c) The torque per voltage takes values that are roughly comparable with an earlier report of torkance coefficients for asymmetric Co-based heterostructures.^30^ Our results suggest that magnetism might be controlled by current-induced SOT in single-molecule junctions. They also suggest that for the studied molecule, the intrinsic SO coupling values are in a range so that SOT-mediated switching may compete with effects related to current-induced magnetic stray fields. Our results present a first step in the way to understanding microscopic SO-induced magnetism, torques, and spin dynamics at the molecular level in tunnel junctions. We believe that our research will stimulate future investigations of spin dynamics (for instance, in terms of the Landau–Lifshitz–Gilbert classical dynamics employing an ab initio parametrization^73^) or the study of SOT in other promising types of molecular complexes, e.g., in chiral molecules for the application in chirality induced spin selectivity (CISS).^74^

## Supporting Information Available

Computational details, technical mathematical derivations of the nonequilibrium density matrix with spin–orbit coupling, details on the DFT-NEGF cycle with spin–orbit coupling, relation between the expressions of the equilibrium and nonequilibrium density matrix, additional computational data of observables not shown in the main text, and junction geometry (PDF)
