# Supplementary material for: Spin–Orbit Torque in Single-Molecule Junctions from ab Initio
Source: J Phys Chem Lett. 2024 May 22;15(21):5747–53. doi: 10.1021/acs.jpclett.4c00502 (PMC11145651; doi:10.1021/acs.jpclett.4c00502)
Supplement: Supplementary file 1 — jz4c00502_si_001.pdf [file jz4c00502_si_001.pdf]

Supporting Information

Spin-orbit torque in single-molecule junctions from *ab initio*

María Camarasa-Gómez,[1,2] Daniel Hernangómez-Pérez,[1,3] and Ferdinand Evers[1]

[1]*Institute of Theoretical Physics, University of Regensburg, 93040 Regensburg, Germany*

[2]*Department of Molecular Chemistry and Materials Science, Weizmann Institute of Science, Rehovot 7610001, Israel*

[3]*CIC nanoGUNE BRTA, Tolosa Hiribidea 76, 20018 San Sebastián, Spain*

Keywords: spin-orbit torque; density functional theory; out-of-equilibrium; single-molecule junctions

Email: maria.camarasa-gomez@weizmann.ac.il

ferdinand.evers@ur.de

CONTENTS

I. Computational details 2

II. Out-of-equilibrium density matrix with spin-orbit coupling 3

III. Relation with the equilibrium density matrix 6

IV. Self-consistent DFT-NEGF cycle with spin-orbit coupling 7

V. Observables 9

VI. Additional results 11

A. Additional figures 11

B. Additional tables 13

C. Junction geometry 13

References 15

I. COMPUTATIONAL DETAILS

Geometry. The geometry for the copper–vanadocene molecular junction was obtained following a well-established two-step process. In the first step, we optimize the coordinates of the molecular atoms, and the molecular atoms with the diamine anchor groups, employing the trust-radius enhanced version of the Broyden-Fletcher-Shanno-Goldfarb algorithm[1]. In a second step, the copper atoms are added and the geometry optimized to obtain the correct contact angle between the molecule and the pyramidal copper tip. Each copper pyramidal cluster used in this work has 11 atoms and was cut in the (111) direction. The geometries are considered to be structurally relaxed once the force components per atoms are smaller than the threshold value 10^{-2} eV/Å.

DFT calculations. For our spin-orbit torque calculations, we employ the FHI-aims package[1]. As exchange-correlation approximation we employ the non-empirical Perdew-Burke-Ernzerhof (PBE) exchange-correlation functional[2]. For simplicity, we avoid the use of hybrid functionals due to inherent problems in describing metallic properties of nanostructures[3]. We include as well scalar relativistic corrections to the kinetic energy employing the atomic zeroth-order regular approximation[4]. The Kohn-Sham states[5] are represented in an all-electron numeric atom-center basis set (see also Sec. II in the Supporting Information). For computational efficiency, we consider the optimized 'light' pre-defined settings. Spin-orbit (SO) coupling is considered as implemented in the FHI-aims code. This code provides the so-called non-self-consistent second-variational method[6], in which, the SO coupling is reintroduced (at first order in $(E - \hat{v})/c^2$, with $\hat{v}$ the scalar potential operator) after a regular SCF calculation with scalar relativistic corrections. The Kohn-Sham Hamiltonian with SO coupling is then rebuilt explicitly and further diagonalized to obtain SO-corrected eigenenergies and eigenstates.

For the density functional theory[7,8] (DFT) calculations, we obtain the ground state of the system using as convergence criteria for the self-consistent field cycle 10^{-6} electrons/Å^3 for the particle density, 10^{-4} eV for the Kohn-Sham eigenvalue sum, and 10^{-7} eV for the total energy.

Transport code. In the self-consistent field cycle shown in the main text (further details in Sec. II), we have interfaced the density functional theory package with

the quantum transport module AITRANSS[9,10], where non-equilibrium Green's function formalism[11,12] (NEGF) is implemented. This package has been extended to incorporate spin-orbit interaction[13].

II. OUT-OF-EQUILIBRIUM DENSITY MATRIX WITH SPIN-ORBIT COUPLING

General definitions. We provide here technical details on the generalization of the non-equilibrium density matrix for finite systems with absorbing boundary conditions[9,10,14]. The spinless case was originally presented in Ref. 10 and it is extended here to non-collinear systems with spin-orbit coupling. The non-equilibrium density matrix is defined within the NEGF from the energy integration of the lesser Green's function, $\hat{G}^{<} := \hat{G}^{<}(E)$,

$$\hat{\rho} = \frac{1}{2\pi i}\int_{-\infty}^{+\infty} dE\hat{G}^{<}, \tag{S1}$$

which in turn can be computed from the Keldysh equation, $\hat{G}^{<} = \hat{G}\hat{\Sigma}^{<}\hat{G}^{\dagger}$. Here, $\hat{G} := \hat{G}(E)$ is the retarded Green's function of the extended molecule

$$\hat{G} = (E\mathbb{1} - \hat{\mathcal{H}}^{\mathrm{KS}} - \hat{\Sigma})^{-1}, \tag{S2}$$

with $\mathbb{1}$ the identity operator, $\hat{\mathcal{H}}^{\mathrm{KS}}$ the Kohn-Sham Hamiltonian and $\hat{\Sigma} := \hat{\Sigma}(E) = \hat{\Sigma}_L(E) + \hat{\Sigma}_R(E)$ the self-energy operator of the left (L) and right (R) leads. The operator $\hat{\Sigma}^{<} := \hat{\Sigma}^{<}(E)$ represents the lesser self-energy and is given by

$$\hat{\Sigma}^{<} = i\left[f(E-\mu_L)\hat{\Gamma}_L(E) + f(E-\mu_R)\hat{\Gamma}_R(E)\right], \tag{S3}$$

with $f(E-\mu_\alpha)$ the Fermi-Dirac distribution function of each reservoir, characterized by chemical potential μ_α, and $\hat{\Gamma} := \hat{\Gamma}(E)$ is the anti-Hermitian part of the self-energy

$$\hat{\Gamma}_\alpha = i\left[\hat{\Sigma}_\alpha - \hat{\Sigma}_\alpha^{\dagger}\right], \tag{S4}$$

with $\alpha \in \{L, R\}$.

Basis set and reconstructed Kohn-Sham Hamiltonian. The Kohn-Sham equations are solved within the FHI-aims package using a numeric atom-center basis set, $\varphi_j(\mathbf{r}) = \langle \mathbf{r}|\varphi_j\rangle := \langle \mathbf{r}|j\rangle$. Each Kohn-Sham spinor $\psi_\mu(\mathbf{r}) = \langle \mathbf{r}|\psi_\mu\rangle := \langle \mathbf{r}|\mu\rangle$, where $\mu = 1, \cdots N_{\text{states}}$ with $N_{\text{states}} = 2N$ and N being the number of spin-resolved orbitals, can be expanded as follows

$$\psi_\mu(\mathbf{r}) = \begin{bmatrix} \psi_\mu^\uparrow(\mathbf{r}) \\ \psi_\mu^\downarrow(\mathbf{r}) \end{bmatrix} = \sum_{j=1}^{N} \begin{pmatrix} c_{\mu j}^\uparrow \\ c_{\mu j}^\downarrow \end{pmatrix} \varphi_j(\mathbf{r}), \tag{S5}$$

where $c_{\mu j}^\sigma$ are the spin-projected Kohn-Sham coefficients. In general, the Kohn-Sham coefficients can be recast into a complex quadratic matrix of dimension $2N \times 2N$, so that Eq. (S5) reads

$$\psi_\mu(\mathbf{r}) = \sum_{\nu=1}^{2N} B_{\nu\mu}\varphi_\nu(\mathbf{r}). \tag{S6}$$

Whenever the set of atom-center orbitals is non-orthogonal, it can be orthogonalized by a Löwdin orthogonalization procedure[15] using the real-valued symmetric overlap matrix

$$S_{\nu\nu'} = \langle \nu|\nu'\rangle = \begin{cases} \langle \varphi_\nu|\varphi_{\nu'}\rangle \text{ if } \nu, \nu' \leq N \text{ and } \sigma = \sigma' =\uparrow, \\ \langle \varphi_{\nu-N}|\varphi_{\nu'-N}\rangle \text{ if } \nu, \nu' \geq N \text{ and } \sigma = \sigma' =\downarrow, \end{cases} \tag{S7}$$

so that the orthogonal basis relates to the non-orthogonal one by

$$|\tilde{\nu}\rangle = \sum_{\nu'=1}^{2N} S_{\nu',\nu}^{-1/2}|\nu'\rangle. \tag{S8}$$

The Kohn-Sham Hamiltonian can therefore be reconstructed from the set of Kohn-Sham spinors and energies, ϵ_μ in a straightforward way

$$\begin{aligned} \mathcal{H}^{\text{KS}} &= \sum_{\mu=1}^{2N} |\psi_\mu\rangle\epsilon_\mu\langle\psi_\mu| = \sum_{\mu=1}^{2N}\sum_{\nu,\nu'=1}^{2N} |\nu\rangle B_{\nu\mu}\epsilon_\mu[B^*]_{\mu\nu'}\langle\nu'|, \\ &= \sum_{\mu=1}^{2N}\sum_{\nu,\nu'=1}^{2N}\sum_{\tilde{\nu},\tilde{\nu}'=1}^{2N} |\tilde{\nu}\rangle S_{\tilde{\nu}\nu}^{1/2} B_{\nu\mu}\epsilon_\mu[B^*]_{\mu\nu'} S_{\nu'\tilde{\nu}'}^{1/2}\langle\tilde{\nu}'|. \end{aligned} \tag{S9}$$

which can be recast in a matrix multiplication form, $\hat{\mathcal{H}}^{\text{KS}} = S^{1/2}B\boldsymbol{\epsilon}B^\dagger S^{1/2}$, where $\boldsymbol{\epsilon}$ is the Kohn-Sham energy (diagonal) matrix.

Markovian out-of-equilibrium density matrix. For simplicity, we assumed in this paper that the leads are closed shell and non-polarized with vanishing spin-orbit interaction. Therefore, the self-energy is block-diagonal in spin space and spin independent, $\hat{\Sigma}_{\alpha}^{\sigma} = \hat{\Sigma}_{\alpha}^{\bar{\sigma}} \equiv \hat{\Sigma}_{\alpha}$, with $\sigma = \{\uparrow, \downarrow\}$ and $\bar{\sigma}$ corresponding to flip σ. The effect of the spin-orbit interaction in the Green's function will thus come from the off-diagonal blocks of the Kohn-Sham Hamiltonian labeled by the spin indices $\sigma, \bar{\sigma}$. Taking advantage of the Markovian approximation considered in the absorbing boundary condition scheme for the self-energy[9,10,14], we perform a rotation of the Green's function to the basis that diagonalizes the complex matrix $\hat{\mathcal{H}}_{\Sigma} = \hat{\mathcal{H}}^{\rm KS} + \mathbb{1}_2 \otimes \hat{\Sigma}$. Because this matrix is non-Hermitian, we choose to work with the right set of eigenvalues and eigenvectors $\hat{\mathcal{H}}_{\Sigma} B = BZ$ where $Z = \mathrm{diag}(Z_1, \ldots, Z_{2N})$, with $Z_{\mu} = \mathrm{Re}Z_{\mu} + i\mathrm{Im}Z_{\mu}$, is the eigenvalue matrix and B the matrix that contains the right eigenvectors in each column. In this basis, the Green's function and the matrix blocks in spin-space are given by

$$\hat{G}^{\sigma\sigma'} = B^{\sigma} \hat{\mathcal{G}} ([B^{-T}]^{\sigma})^{T}, \tag{S10}$$

with $B^{-T} = (B^{-1})^{T}$. Here, $\hat{\mathcal{G}} := \hat{\mathcal{G}}(E) = [E\mathbb{1} - Z]^{-1}$ and B^{σ} corresponds to a $N \times 2N$ matrix extracted from B by projecting the eigenvectors into the spin subspace labeled by σ. Using Eq. (S10), the lesser Green's function reads

$$G^{<,\sigma,\sigma'} = \sum_{\sigma_1} B^{\sigma} \hat{\mathcal{G}} ([B^{-T}]^{\sigma_1})^{T} \hat{\Sigma}^{<,\sigma_1} [(B^{-1})^{\dagger}]^{\sigma_1} \hat{\mathcal{G}}^{*} (B^{\sigma'})^{\dagger}. \tag{S11}$$

We substitute Eq. (S11) into Eq. (S1) and use Eq. (S3) in the zero-temperature limit, in which the Fermi-Dirac distribution reduces to the Heaviside step function, $f(E - \mu_{\alpha}) \rightarrow \theta(E - \mu_{\alpha})$ to find

$$\rho^{\sigma\sigma'} = \sum_{\alpha} B^{\sigma} \left[\frac{1}{2\pi} \int_{-\infty}^{\mu_{\alpha}} dE \, \hat{\mathcal{G}} \hat{\tilde{\Gamma}}_{\alpha} \hat{\mathcal{G}}^{*} \right] (B^{\sigma'})^{\dagger}, \tag{S12}$$

where we have defined $\hat{\tilde{\Gamma}}_{\alpha} := \sum_{\sigma} ([B^{-T}]^{\sigma})^{T} \hat{\Gamma}_{\alpha}^{\sigma} ([B^{-1}]^{\dagger})^{\sigma}$. We can recast this expression into a familiar form by writing $\rho^{\sigma\sigma'} = B^{\sigma} \hat{J} (B^{\sigma'})^{\dagger}$ where the matrix $\hat{J} := \sum_{\alpha} \hat{J}_{\alpha}$ is given by

$$\hat{J}_{\alpha} = \frac{1}{2\pi} \int_{-\infty}^{\mu_{\alpha}} dE \, \hat{\mathcal{G}}(E) \hat{\tilde{\Gamma}}_{\alpha} \hat{\mathcal{G}}^{*}(E). \tag{S13}$$

This integral has been evaluated analytically using contour integration[10], where its matrix elements are given by $(J_\alpha)_{\mu\nu} = (\tilde{\Gamma}_\alpha)_{\mu\nu} F_{\mu\nu}(\mu_\alpha)$ with $\mu, \nu = 1, \ldots, 2N$,

$$F_{\mu\nu}(\mu) = \frac{1}{2\pi} \frac{1}{(\epsilon_\mu - \epsilon_\nu) + i(\eta_\mu - \eta_\nu)} \Big[-2\pi i + \frac{1}{2} \ln\left(\frac{\epsilon_\mu^2 + \eta_\mu^2}{\epsilon_\nu^2 + \eta_\nu^2}\right) - i\arctan\left(\frac{\eta_\mu}{\epsilon_\mu}\right) - i\arctan\left(\frac{\eta_\nu}{\epsilon_\nu}\right) \Big], \quad \text{(S14)}$$

and $\epsilon_\mu = \mu - \mathrm{Re}Z_\mu$ and $\eta_\mu = \mathrm{Im}Z_\mu$. The matrix $\hat{J}$ is simply the non-equilibrium generalization of the occupation numbers at equilibrium, as shown in Sec. III.

III. RELATION WITH THE EQUILIBRIUM DENSITY MATRIX

The general form of the density operator of a quantum system defined in a finite-dimensional Hilbert space is given by[16,17]

$$\hat{\rho} = \sum_\mu f_\mu \left|\psi_\mu\right\rangle \left\langle\psi_\mu\right|, \quad \text{(S15)}$$

where the (fractional) occupations are given by $0 \le f_\mu \le 1$ and the sum runs over all the available states, $\mu = 1, \ldots, N_{\text{states}}$. At equilibrium, the quantum state $|\psi_\mu\rangle$ is a Kohn-Sham state, which in the presence of spin-orbit interaction is represented as a two-component spinor

$$|\psi_\mu\rangle = \sum_\sigma |\sigma\rangle \otimes |\psi_\mu^\sigma\rangle \equiv \begin{bmatrix} |\psi_\mu^\uparrow\rangle \\ |\psi_\mu^\downarrow\rangle \end{bmatrix}, \quad \text{(S16)}$$

where we remind that $|\psi_\mu^\sigma\rangle = \sum_{j=1}^N c_{\mu j}^\sigma |\varphi_j\rangle$ is expanded in the basis of spin-resolved Kohn-Sham orbitals ($N_{\text{states}} = 2N$), similar to Eq. (S5). Using Eqs. (S15) and (S16), the spin-resolved blocks of the equilibrium density matrix in the presence of SO interaction can be expressed in the following way

$$\rho^{\sigma\sigma'} = c^\sigma \mathfrak{f} (c^{\sigma'})^\dagger. \quad \text{(S17)}$$

Here, c^σ are $N \times 2N$ matrices with the spin-resolved Kohn-Sham coefficients at equilibrium (analogous to the B^σ matrices for the open quantum system) and $\mathfrak{f}$ is a diagonal matrix of

dimension $2N \times 2N$ in which the occupation numbers are in the diagonal, *i.e.* $\mathfrak{f}_{\mu\nu} = f_\mu \delta_{\mu\nu}$. Comparison with the non-equilibrium expression derived in Sec. II, $\rho^{\sigma\sigma'} = B^\sigma \hat{J}(B^{\sigma'})^\dagger$, yields the interpretation of J as the non-equilibrium generalization of the occupation numbers at equilibrium.

IV. SELF-CONSISTENT DFT-NEGF CYCLE WITH SPIN-ORBIT COUPLING

In this section we provide additional details on the implementation and physics of the self-consistent DFT-NEGF cycle[10,13] presented in the main text. This self-consistent cycle explicitly includes the effect of SO coupling, compared to available tools in the literature[10,18–22], which do not incorporate the effect of SO interaction. In this regard, also our DFT-NEGF self-consistent cycle extends previous works in which the non-equilibrium density matrix with SO coupling is calculated at first order in the linear voltage and in a non-self-consistent way[23–25].

Density-matrix update. The self-consistent DFT-NEGF cycle requires to update the Kohn-Sham Hamiltonian at every step, which itself is a functional of the density matrix. We choose for the update to employ the blocks $\text{Re}\,[\hat{\rho}^{\sigma\sigma}]$. In other words, it is enough to update the densities, as DFT physical properties are indeed a functional of the particle density[26,27].

Self-energy and charge neutrality condition. Although in principle one could compute the exact self-energy of the semi-infinite lead, see *e.g.* Refs. 18 and 28, here we employ instead the computationally advantageous absorbing boundary condition scheme[9,10,14], as mentioned in Sec. II. Within the this scheme, the self-energy can be written as

$$\hat{\Sigma}_\alpha = \sum_{\tilde{\mu},\tilde{\nu} \in \mathcal{S}_\alpha} |\tilde{\mu}\rangle\, [\delta\epsilon - i\eta] \delta_{\tilde{\mu}\tilde{\nu}}\, \langle\tilde{\mu}|\,, \tag{S18}$$

where the real part, $\delta\epsilon_\sigma$, corresponds to a compensating energy shift and the imaginary part, η, to a local absorption rate, both only active in the subspace of atoms that belong to the outermost external layers of the metallic cluster representing the electrodes, $\mathcal{S}_\alpha$.

The absorption rate, η, is a parameter which is chosen so that physical quantities for a given system (density of states, transmission function ...) are stable and almost invariant

under moderate changes in the value for η (here, we consider $\eta = 0.05$ Ha). In addition, from Eq. (S12) it is clear that the non-equilibrium density matrix has a parametric dependence on $\delta\epsilon$ and E_{F}, the latter understood as $\mu_L = \mu_R := E_{\mathrm{F}}$ at zero bias. First, using an initial guess for E_{F} and assuming that screening occurs within our extended molecule boundaries, we fix the value for $\delta\epsilon$ given a Fermi energy, also ensuring the charge neutrality within some tolerance

$$|N_{\mathrm{elec}} - \langle N \rangle| \leq \zeta N_{\mathrm{elec}}, \tag{S19}$$

and

$$\langle N \rangle = \mathrm{Tr}\left[\hat{\rho}(\delta\epsilon)\right] = \sum_{i=1}^{N}\sum_{\sigma} \rho_{ii}^{\sigma\sigma}(\delta\epsilon_n). \tag{S20}$$

A reasonable value for this tolerance parameters is $\zeta = 10^{-4}$. The convergence of this self-consistent cycle produces $\hat{\rho}(\delta\epsilon_\infty)$.

Next, we correct for nonphysical charge accumulation at the boundaries of the metal cluster given the optimal $\delta\epsilon_\infty$, by minimizing the external charge at the interface

$$\delta Q^{\mathrm{outer}} = \sum_{i\in\mathcal{S}_\alpha}\sum_{\sigma} \rho_{ii}^{\sigma\sigma} - Q^{\mathrm{ref}}, \tag{S21}$$

where $Q^{\mathrm{ref}} = N_{\mathcal{S}_\alpha} Z_{\mathcal{S}_\alpha}$, being $N_{\mathcal{S}_\alpha}$ the number of atoms of the contact region and $Z_{\mathcal{S}_\alpha}$ is the total charge per atom. We compute these charges by using a Löwdin population analysis. In precedent studies[10], it has been proven that $\delta Q^{\mathrm{outer}}$ is a linear function of E_{F} (and therefore $\delta\epsilon_\infty$). The optimized self-energy is obtained for $\delta Q^{\mathrm{outer}}(\delta\epsilon^*) = 0$, which fixes $\delta\epsilon^*$ and the corresponding Fermi energy $E_{\mathrm{F}}^*(\delta\epsilon^*)$ for any ensuing non-equilibrium calculation.

Finite bias. Once the self-energy is properly parametrized, finite bias in the system can be considered by setting a difference in the chemical potentials of the two reservoirs, $\mu_L - \mu_R = eV_{\mathrm{bias}}$ with $\mu_L > \mu_R$ by construction. We note that, within this scheme, the reference chemical potential, $\bar{\mu} = (\mu_L + \mu_R)/2$, which corresponds to the Fermi energy at zero bias, is not fixed. Therefore, we can still adjust the value to make sure that the charge neutrality condition is satisfied at finite bias within a given precision, N_ζ. In practical terms, we consider the finite bias calculation to be converged if $|\mathrm{Tr}[\hat{\rho}(\bar{\mu})] - N_{\mathrm{elec}}| < N_\zeta$ with $N_\zeta = 10^{-7}$ electrons.

Magnetic anisotropy term. As explained in the main text, if the magnetic anisotropy energy is too small compared to the voltage window, the spin density will oscillate in the self-consistent cycle at finite bias. A possible solution to this scenario is to perform a "magnetic stabilization" of the spin density towards the easy magnetization axis of the system. This is done by adding a local magnetic anisotropy term in the atom where most of the spin density (or spin magnetic moment) is located. In practical terms, we add a term to the Kohn-Sham Hamiltonian in the step where we build the Kohn-Sham Hamiltonian to incorporate it into the non-equilibrium density matrix, see Eq. (S2). In the molecular orbital basis, the term reads

$$\Sigma_{\mathrm{MAE}}^{\sigma\sigma'} = \sigma\Delta\delta_{\sigma,\sigma'} \sum_{p\in\mathcal{A}} \sum_{j\in\mathcal{O}_{\mathcal{A}}} |p,j\rangle \langle p,j| \,. \tag{S22}$$

Here, Δ is a parameter and $\mathcal{A}$ is the set of atoms where the term is non-zero (with corresponding orbitals $\mathcal{O}_{\mathcal{A}}$.

Exchange-correlation field. The components of the exchange-correlation magnetic field, formally obtained as the functional derivative of the XC energy with respect to the magnetization, can be computed from the spin-blocks of the Kohn-Sham Hamiltonian as

$$B_{\mathrm{xc}}^{x} = (\hat{\mathcal{H}}^{\mathrm{KS}})^{\uparrow\downarrow} + (\hat{\mathcal{H}}^{\mathrm{KS}})^{\downarrow\uparrow}, \tag{S23}$$

$$B_{\mathrm{xc}}^{y} = i[(\hat{\mathcal{H}}^{\mathrm{KS}})^{\uparrow\downarrow} - (\hat{\mathcal{H}}^{\mathrm{KS}})^{\downarrow\uparrow}], \tag{S24}$$

$$B_{\mathrm{xc}}^{z} = (\hat{\mathcal{H}}^{\mathrm{KS}})^{\uparrow\uparrow} - (\hat{\mathcal{H}}^{\mathrm{KS}})^{\downarrow\downarrow}. \tag{S25}$$

V. OBSERVABLES

After convergence of the self-consistent DFT-NEGF cycle (see main text), we can proceed with the calculation of physical observables or other magnitudes of interest. For example, from the knowledge of the self-consistent Green's function at finite bias, we can evaluate the finite bias transmission function

$$T(E,V_{\mathrm{bias}}) = \mathrm{Tr}\left[\hat{\Gamma}_L \hat{G}(E,V_{\mathrm{bias}})\Gamma_R \hat{G}^{\dagger}(E,V_{\mathrm{bias}})\right]. \tag{S26}$$

It can be shown straightforwardly that the transmission function can be written as a sum over the spin-resolved (spin-conserving and spin-flip) components

$$T(E, V_{\text{bias}}) = \sum_{\sigma,\sigma'} T_{\sigma,\sigma'}(E, V_{\text{bias}}), \tag{S27}$$

the latter adopting a particularly simple form in the basis in which the matrix $\hat{H}_\Sigma$ is diagonal

$$T_{\sigma,\sigma'}(E, V_{\text{bias}}) = \text{Tr}\left[\tilde{\Gamma}_L^\sigma \hat{\mathcal{G}} \tilde{\Gamma}_R^{\sigma'} \hat{\mathcal{G}}^*\right]_{V_{\text{bias}}}. \tag{S28}$$

Here, we have defined the rotated anti-Hermitian part of the left/right self-energy as

$$\tilde{\Gamma}_L^\sigma = (B^\sigma)^\dagger \Gamma_L B^\sigma, \tag{S29a}$$

$$\tilde{\Gamma}_R^\sigma = ([B^{-T}]^\sigma)^T \Gamma_R [(B^{-1})^\dagger]^\sigma, \tag{S29b}$$

and we recall that $\hat{\mathcal{G}} := \hat{\mathcal{G}(E)}$ is the Green's function in the right eigenbasis of $\hat{\mathcal{H}}_\Sigma$. Finally, the charge current can be expressed as[11,29]

$$I(E, V_{\text{bias}}) = \frac{2e}{h} \int_{\infty}^{+\infty} dE \left[f(E - \mu_L) - f(E - \mu_R)\right] T(E, V_{\text{bias}}). \tag{S30}$$

Other observables are obtained by a trace of the non-equilibrium density matrix at fixed bias with the corresponding operator, see also main text.

VI. ADDITIONAL RESULTS

A. Additional figures

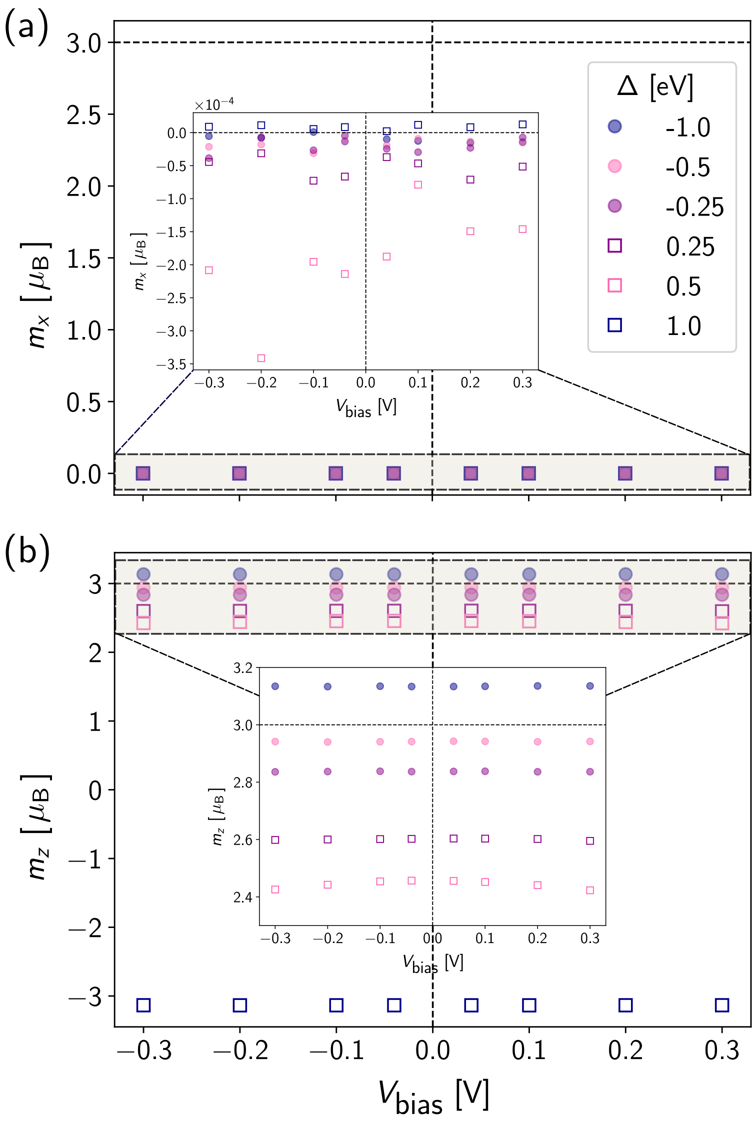

Fig. S1. Magnetization, m_α, at the vanadium atom as a function of the voltage bias, V_{bias}, applied across the molecular junction. We display the traces obtained for several representative values of the magnetic anisotropy term in the two relevant spatial directions (a) $\hat{\mathbf{x}}$ and (b) $\hat{\mathbf{z}}$. The dashed horizontal line represents the value expected for the local magnetization in the isolated vanadocene molecule, oriented in the $\hat{\mathbf{z}}$ direction. The inset in each panel corresponds to a zoom of the data inside the dashed light beige rectangle.

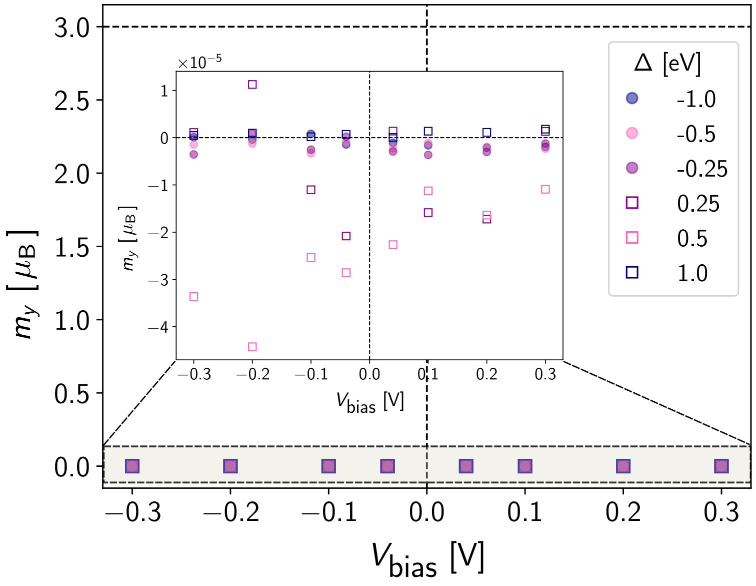

Fig. S2. Magnetization, m_y at the vanadium atom as a function of the voltage bias, V_{bias}, applied across the molecular junction. The dashed horizontal line represents the value expected for the local magnetization in the isolated vanadocene molecule, directed in the $\hat{\mathbf{z}}$ direction, shown here as comparison. The inset corresponds to a zoom of the data inside the dashed light brown rectangle.

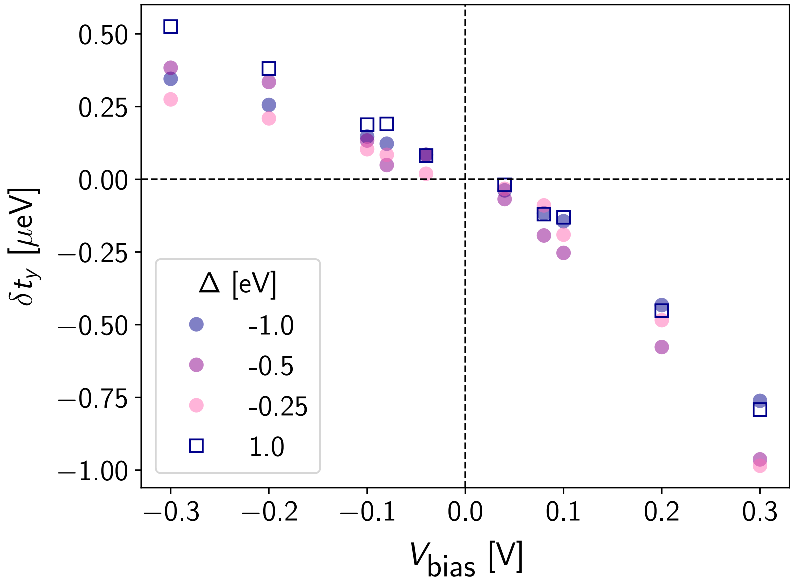

Fig. S3. Local SO torque response, δt_y, exerted at the spin located in the vanadium atom as a function of the voltage bias, V_{bias}, applied across the molecular junction.

B. Additional tables

	$\Delta = 0$ eV		$\Delta = -1.0$ eV	
	No SO coupling	SO coupling	No SO coupling	SO coupling
HOMO-1	-3.7168	-3.7187	-3.4979	-3.4997
HOMO	-3.5860	-3.5737	-3.3572	-3.3552
LUMO	-1.8668	-1.8676	-1.6289	-1.6294
LUMO+1	-1.6314	-1.6311	-1.3698	-1.3696

TABLE I. Kohn-Sham energies of the most representative molecular orbitals of the isolated vanadocene molecule. All energies are given in eV. We show the values for two representative cases $\Delta = 0$ and $\Delta = -1.0$ eV. The level splitting due to SO coupling is of the order of meV. Concerning the magnetic anisotropy terms under consideration here, the shifts in the Kohn-Sham energies are an order of magnitude smaller. These shifts do not influence the relative ordering of the energy levels.

C. Junction geometry

```
atom -3.31867693 -0.06187045 4.35673893 Cu
atom -5.20228950 0.72186008 5.79267608 Cu
atom -2.88558216 0.72993814 6.64372828 Cu
atom -4.09535943 -1.41077248 6.24771781 Cu
atom -2.27267900 -2.89247000 8.96326200 Cu
atom -7.08737900 1.53856800 7.16908800 Cu
atom -4.67552700 1.54376600 8.04046800 Cu
atom -2.26367600 1.54896400 8.91184800 Cu
atom -5.88595400 -0.67955000 7.63048500 Cu
atom -3.47410400 -0.67435200 8.50186500 Cu
atom -4.68453000 -2.89766800 8.09188200 Cu
atom -2.65423855 -0.33020629 2.36545816 N
atom -3.12384327 -1.18682727 2.05423888 H
atom -3.08850991 0.43767169 1.84247646 H
atom -2.58445402 -0.84769963 -1.78526729 H
```

atom -0.65214719 1.75915198 1.95730800 H

atom -1.51811663 -0.64597132 -1.84818991 C

atom -1.24285835 -0.40516733 2.05183247 C

atom -0.36476566 0.71081090 1.92292851 C

atom -0.63615589 -2.70993339 -2.04454787 H

atom -1.40634938 1.59613252 -1.76495526 H

atom -0.49063183 -1.63397809 -1.98050888 C

atom -0.90029606 0.63736550 -1.84383017 C

atom -0.19691969 -0.49224728 -0.01553840 V

atom -0.47001932 -1.60175256 1.96908726 C

atom 0.96639067 0.20188917 1.79667540 C

atom -0.85066276 -2.61744856 2.05066946 H

atom 1.86585139 0.80528680 1.70418569 H

atom 0.75577010 -0.94761530 -2.08095580 C

atom 0.51233728 0.45304829 -1.97646388 C

atom 0.90284977 -1.22043125 1.82595422 C

atom 1.26155242 1.23968547 -2.02757459 H

atom 2.03096840 -1.55757029 -2.39009416 N

atom 2.78415331 -1.05519807 -1.90829725 H

atom 2.05185165 -2.51665718 -2.02791050 H

atom 1.74653248 -1.90400741 1.77309523 H

atom 2.69567186 -1.73940944 -4.38823462 Cu

atom 2.19661522 -1.46467877 -6.75320968 Cu

atom 4.53432536 -1.42684065 -6.02779276 Cu

atom 3.29165075 -3.55646920 -5.97407617 Cu

atom 0.18267600 -3.41980600 -9.00216500 Cu

atom 1.47362300 -1.20480200 -9.06190000 Cu

atom 3.93188400 -1.13342100 -8.33511500 Cu

atom 6.39014400 -1.06204100 -7.60832900 Cu

atom 2.64093700 -3.34842600 -8.27538000 Cu

atom 5.09919600 -3.27704500 -7.54859500 Cu

atom 3.80824900 -5.49204900 -7.48886000 Cu

[1] V. Blum, R. Gehrke, F. Hanke, P. Havu, V. Havu, X. Ren, K. Reuter, and M. Scheffler, "Ab initio molecular simulations with numeric atom-centered orbitals," Computer Physics Communications **180**, 2175 – 2196 (2009).

[2] J. P. Perdew, K. Burke, and M. Ernzerhof, "Generalized gradient approximation made simple," Phys. Rev. Lett. **77**, 3865–3868 (1996).

[3] W. Gao, T. A. Abtew, T. Cai, Y.-Y. Sun, S. Zhang, and P. Zhang, "On the applicability of hybrid functionals for predicting fundamental properties of metals," Solid State Communications **234-235**, 10–13 (2016).

[4] E. van Lenthe, J. G. Snijders, and E. J. Baerends, "The zero-order regular approximation for relativistic effects: The effect of spin–orbit coupling in closed shell molecules," The Journal of Chemical Physics **105**, 6505–6516 (1996).

[5] W. Kohn and L. J. Sham, "Self-consistent equations including exchange and correlation effects," Phys. Rev. **140**, A1133–A1138 (1965).

[6] W. P. Huhn and V. Blum, "One-hundred-three compound band-structure benchmark of post-self-consistent spin-orbit coupling treatments in density functional theory," Phys. Rev. Mater. **1**, 033803 (2017).

[7] M. Dreizler and E. K. U Gross, *Density Functional Theory: An Approach to the Quantum Many-Body Problem*, 1st ed. (Springer, Berlin, 1990).

[8] R. G. Parr and W. Yang, *Density Functional Theory of Atoms and Molecules*, 1st ed. (Oxford University Press, Oxford, 1989).

[9] A. Arnold, F. Weigend, and F. Evers, "Quantum chemistry calculations for molecules coupled to reservoirs: Formalism, implementation, and application to benzenedithiol," The Journal of Chemical Physics **126**, 174101 (2007).

[10] A. Bagrets, "Spin-polarized electron transport across metal–organic molecules: A density functional theory approach," J. Chem. Theory Comput. **9**, 2801–2815 (2013).

[11] M. Di Ventra, *Electrical Transport in Nanoscale Systems*, 1st ed. (Cambridge University Press, Cambridge, 2008).

[12] J. C. Cuevas and E. Scheer, *Molecular Electronics*, 2nd ed. (World Scientific, 2010).

[13] M. Camarasa-Gómez, *Ab Initio Electronic Transport in Single-Molecule Junctions: Quantum Interference Effects and Spin-Orbit Torque*, Ph.D. thesis, Universität Regensburg (2021).

[14] F. Evers and A. Arnold, "Molecular conductance from ab initio calculations: Self energies and absorbing boundary conditions," in *CFN Lectures on Functional Nanostructures - Vol. 2, Lecture Notes in Physics*, Vol. 820, edited by C. Röthig, G. Schön, and M. Vojta (Springer, 2011) pp. 27–53.

[15] P.-O. Löwdin, "On the non-orthogonality problem connected with the use of atomic wave functions in the theory of molecules and crystals," The Journal of Chemical Physics **18**, 365–375 (1950).

[16] J. J. Sakurai and J. Napolitano, *Modern quantum mechanics*, 3rd ed. (Cambridge University Press, Cambridge, 2021).

[17] H. P. Breuer and F. Petruccione, *The theory of open quantum systems*, 1st ed. (Oxford University Press, New York, 2002).

[18] A. R. Rocha, V. M. García-Suárez, S. Bailey, C. Lambert, J. Ferrer, and S. Sanvito, "Spin and molecular electronics in atomically generated orbital landscapes," Phys. Rev. B **73**, 085414 (2006).

[19] N. Papior, N. Lorente, T. Frederiksen, A. García, and M. Brandbyge, "Improvements on non-equilibrium and transport Green function techniques: The next-generation transiesta," Computer Physics Communications **212**, 8–24 (2017).

[20] W. Dednam, C. Sabater, O. Tal, J. J. Palacios, A. E. Botha, and M. J. Caturla, "Refined electron-spin transport model for single-element ferromagnetic systems: Application to nickel nanocontacts," Phys. Rev. B **102**, 245415 (2020).

[21] B.-H. Huang, Y.-F. Lai, and Y.-H. Tang, "Validity of dft-based spin-orbit torque calculation for perpendicular magnetic anisotropy in iron thin films," AIP Advances **13**, 015034 (2023).

[22] B.-H. Huang, Y.-H. Fu, C.-C. Kaun, and Y.-H. Tang, "Determining perpendicular magnetic anisotropy in Fe/MgO/Fe magnetic tunnel junction: A DFT-based spin–orbit torque method," Journal of Magnetism and Magnetic Materials **585**, 171098 (2023).

[23] B. K. Nikolić, K. Branislav, Kapildeb Dolui, Marko D. Petrović, Petr Plecháč, Troels Markussen, and Kurt Stokbro, "First-principles quantum transport modeling of spin-transfer and spin-orbit torques in magnetic multilayers," in *Handbook of Materials Modeling: Applications: Current and Emerging Materials*, edited by Wanda Andreoni and Sidney Yip (Springer International

Publishing, 2018) pp. 1–35.

[24] K. D. Belashchenko, Alexey A. Kovalev, and M. van Schilfgaarde, "First-principles calculation of spin-orbit torque in a Co/Pt bilayer," Phys. Rev. Mater. **3**, 011401 (2019).

[25] K. Dolui, M. D. Petrović, K. Zollner, P. Plecháč, J. Fabian, and B. K. Nikolić, "Proximity spin–orbit torque on a two-dimensional magnet within van der Waals heterostructure: Current-driven antiferromagnet-to-ferromagnet reversible nonequilibrium phase transition in bilayer CrI_3," Nano Letters **20**, 2288–2295 (2020).

[26] R. O. Jones, "Density functional theory: Its origins, rise to prominence, and future," Rev. Mod. Phys. **87**, 897–923 (2015).

[27] F. Evers, R. Korytár, S. Tewari, and J. M. van Ruitenbeek, "Advances and challenges in single-molecule electron transport," Rev. Mod. Phys. **92**, 035001 (2020).

[28] M. Brandbyge, J.-L. Mozos, P. Ordejón, J. Taylor, and K. Stokbro, "Density-functional method for nonequilibrium electron transport," Phys. Rev. B **65**, 165401 (2002).

[29] M. Büttiker, Y. Imry, R. Landauer, and S. Pinhas, "Generalized many-channel conductance formula with application to small rings," Phys. Rev. B **31**, 6207–6215 (1985).
